# Radiomics in predicting mutation status for thyroid cancer: A preliminary study using radiomics features for predicting BRAF^V600E^ mutations in papillary thyroid carcinoma

**DOI:** 10.1371/journal.pone.0228968

**Published:** 2020-02-13

**Authors:** Jung Hyun Yoon, Kyunghwa Han, Eunjung Lee, Jandee Lee, Eun-Kyung Kim, Hee Jung Moon, Vivian Youngjean Park, Kee Hyun Nam, Jin Young Kwak

**Affiliations:** 1 Department of Radiology, Severance Hospital, Research Institute of Radiological Science, Yonsei University, College of Medicine, Seoul, Korea; 2 Department of Radiology, Research Institute of Radiological Science and Center for Clinical Imaging Data Science, Yonsei University, College of Medicine, Seoul, Korea; 3 Department of Computational Science and Engineering, Yonsei University, College of Medicine, Seoul, Korea; 4 Department of Surgery, Yonsei University, College of Medicine, Seoul, Korea; Johns Hopkins University, UNITED STATES

## Abstract

**Purpose:**

To evaluate whether if ultrasonography (US)-based radiomics enables prediction of the presence of BRAF^V600E^ mutations among patients diagnosed as papillary thyroid carcninoma (PTC).

**Methods:**

From December 2015 to May 2017, 527 patients who had been treated surgically for PTC were included (training: 387, validation: 140). All patients had BRAF^V600E^ mutation analysis performed on surgical specimen. Feature extraction was performed using preoperative US images of the 527 patients (mean size of PTC: 16.4mm±7.9, range, 10–85 mm). A Radiomics Score was generated by using the least absolute shrinkage and selection operator (LASSO) regression model. Univariable/multivariable logistic regression analysis was performed to evaluate the factors including Radiomics Score in predicting BRAF^V600E^ mutation. Subgroup analysis including conventional PTC <20-mm (n = 389) was performed (training: 280, validation: 109).

**Results:**

Of the 527 patients diagnosed with PTC, 428 (81.2%) were positive and 99 (18.8%) were negative for BRAF^V600E^ mutation. In both total 527 cancers and 389 conventional PTC<20-mm, Radiomics Score was the single factor showing significant association to the presence of BRAF^V600E^ mutation on multivariable analysis (all *P*<0.05). C-statistics for the validation set in the total cancers and the conventional PTCs<20-mm were lower than that of the training set: 0.629 (95% CI: 0.516–0.742) to 0.718 (95% CI: 0.650–0.786), and 0.567 (95% CI: 0.434–0.699) to 0.729 (95% CI: 0.632–0.826), respectively.

**Conclusion:**

Radiomics features extracted from US has limited value as a non-invasive biomarker for predicting the presence of BRAF^V600E^ mutation status of PTC regardless of size.

## Introduction

During the past decade, the incidence of thyroid cancer has rapidly increased worldwide, regardless of the demographic groups [1–3]. The majority of thyroid cancers that are being newly detected are papillary thyroid cancers (PTC) [4,5], a subtype consisting of more than 80% of all differentiated thyroid carcinomas [5,6]. In general, PTC is known to have excellent patient outcomes, 5-year survival rates approaching 98–99% [7,8], but approximately 10–15% of patients had aggressive tumor behavior, local recurrence/distant metastasis after treatment or mortality [9–12]. At present, even with the well-known poor prognostic factors such as age over 45 years, male gender, radioactive iodine resistance [9], it is difficult to descriminate to predict which patient has more aggressive forms of PTCs, and effort has been made using various biomarkers in predicting PTC patients with poor outcome.

With the advancement in molecular genetics, various genetic alterations have been revealed and used as an adjunctive diagnostic method or for predicting patient prognosis [8,13,14]. BRAF^V600E^ mutation, the most frequent oncogene in PTC, has been reported to be associated with aggressive clinical features such as large tumor size, extrathyroidal extension and presence of lymph node metastasis [8,14–16], leading to recurrence or mortality. But even with the ability of either mutation in detecting aggressive cancer types, genetic analysis requires specimen tissue for analysis, mostly obtained from invasive surgical procedures. Aside from the information obtained from conventional imaging, radiomics, using data extracted from medical images converted into high-dimensional, mineable, and quantitative imaging features has been applied to revealing tumor physiology. Other studies have linked imaging features to molecular properties of tumors among various organs [17–20], but to the best of our knowledge, no studies have applied radiomics in predicting molecular status of thyroid cancer that can be used in predicting tumor aggressiveness. Based on this, we evaluated whether if ultrasonography (US)-based radiomics enables prediction of the presence of BRAF^V600E^ mutations among patients diagnosed as PTC.

## Materials & methods

This retrospective study has been approved by the institutional review board (IRB) of Severance Hospital, Yonsei University (approval number: 4-2018-0172), with a waiver for patient consent due to the retrospective study design. Signed informed consent was obtained from all patients prior to biopsy or surgical procedures. Images used for data extraction were fully anonymized before data processing according to the instructions of our IRB.

### Patients

We included 527 patients who had been treated surgically with cytologically-proven or suspicious thyroid cancer between December 2015 to May 2017 at Severance Hospital, Seoul, Korea. All patients had BRAF^V600E^ mutation analysis performed on surgical specimen. The 387 consecutive patients who had surgery from December 2015 to December 2016 were used as the training cohort: 300 women, 87 men, mean age, 42.1 years±14.0 (range, 15–82 years). The 140 consecutive patients who had surgery from January 2017 to May 2017 were used as the validation cohort: 105 women, 35 men, mean age, 41.3 years±13.4 (range, 15–74 years). Mean age of the 527 patients were 41.9 years±13.8 (range, 15–82 years). Mean size of the thyroid masses was 16.4 mm±7.9 (range, 10–85 mm).

As the frequency of BRAF^V600E^ mutation has been reported to be associated with tumor size and conventional PTC [21], subgroup analysis was performed including thyroid cancers confirmed as conventional PTCs measuring <20-mm (n = 389). Mean age of the 389 patients was 42.9 years±13.1 (range, 15–80 years). Mean size of the conventional PTCs was 14.9 mm±4.6 (range, 10–19 mm). Clinicopathologic data regarding tumor size, lymph node metastasis were obtained from review of medical records. Imaging features of the thyroid masses used for analysis were obtained from an institutional database.

### US image selection and feature extraction

**Fig 1:**
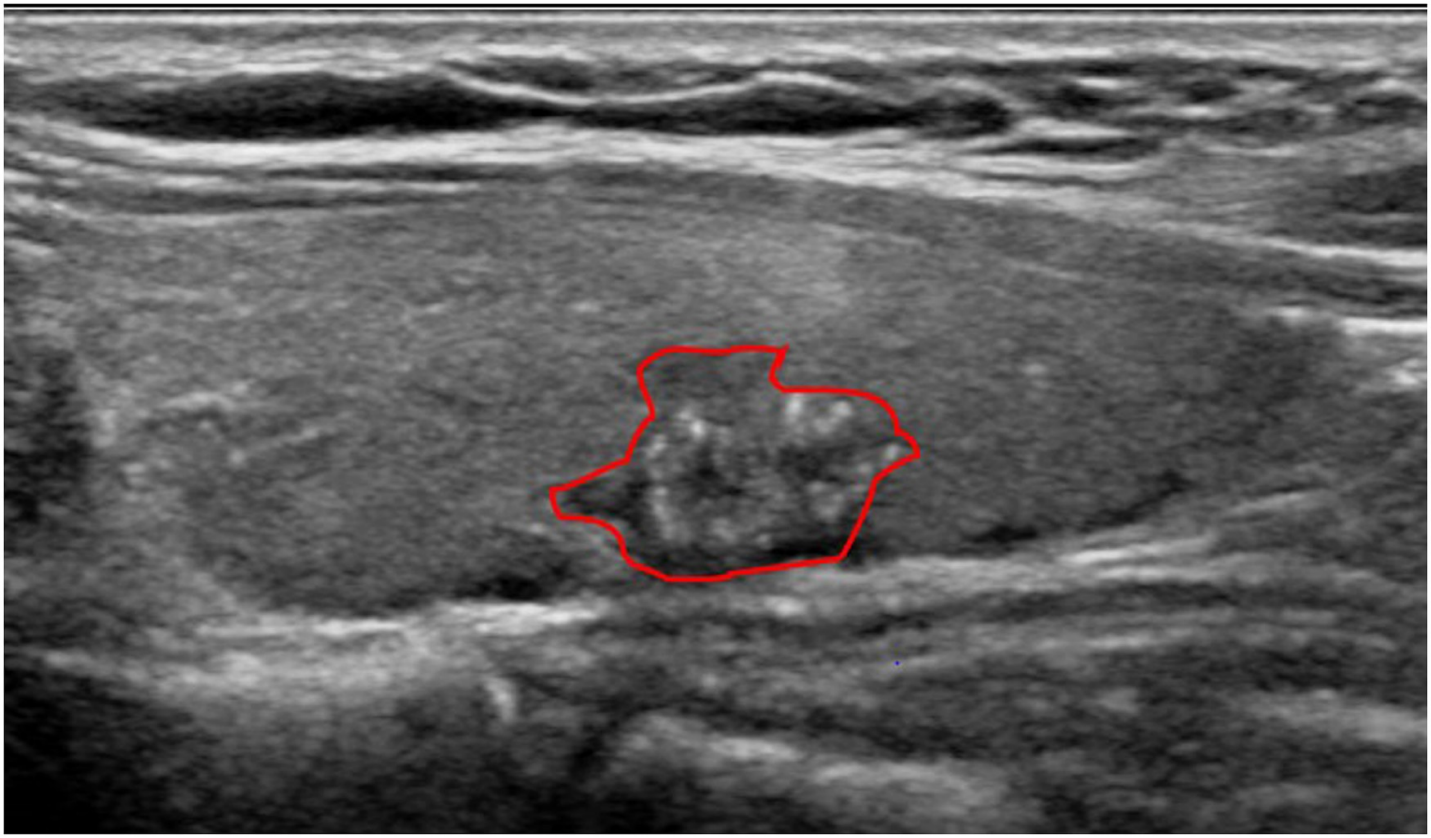
Representative image of tumor segmentation using thyroid US. A diagonal region-of-interest (ROI) was drawn along the tumor border (red line) for feature extraction.

One radiologist (J.Y.K.) reviewed the preoperative US examinations of the 527 patients on the picture archiving and communication system (PACS) and selected representative transverse or longitudinal images of the tumor. The selected representative images were converted into JPEG files for manual segmentation. One radiologist (J.H.Y.) who had 9 years of experience in thyroid imaging manually set a region-of-interest (ROI) along the boundary of the selected tumor using Paint software of Windows (Fig 1). Since ROI marking with colored brush using Paint software alters original intensities in image, the manual ROI segmentation is conducted over the duplicate images of collected JPEG files. Before starting ROI extracting procedure, all images were normalized for fair comparison. First the location information of ROI marking (coordinate information of red curves in Fig 1) was sought and then applied to the original JPEG image to extract ROI only. This procedure ensures that the original intensity of the image was not affected by the ROI extraction process. Once ROI was extracted, a total of 730 feature information were gathered. The 730 features include the first order statistics (energy, entropy, kurtosis, skewness and so on), the second order statistics (the gray level co-occurrence matrix (GLCM) and gray level run-length matrix (GLRLM) were established and the corresponding features were extracted), and features from four discrete one-level wavelet decompositions. The detailed calculation for these features can be found in [22]. To obtain the feature quantities, the house code in MATLAB 2018b was used. Here, 256 bins using a bin with of 1 were utilized for intensity histogram and 4 angles of 0, 45, 90, and 135 degrees were utilized for GLCM and GLRLM anaylsis.

### BRAF^V600E^ mutational analysis

Direct DNA sequencing was used for the surgical specimen in mutation analysis. Exon 15, which contains the BRAF^V600E^ mutation, was amplified by PCR with the foward primer AGGAAAGCATCTCACCTCATC and the reverse primer GATCACACCTGCCTTAAATTGC. The PCR parameters were as follows: 94°C for 5 minutes, 35 cycles at 94°C for 0.5 minutes, 60°C for 0.5 minutes, and 72°C for 10 minutes. The amplified products were purified with a QIAGEN PCR purification kit and sequenced using the foward primer described previously with Big Dye Terminator (ABI Systems, Applied Biosystems, Foster City, CA), and an ABI PRISM 3100 Avant Genetic Analyzer (Perkin-Elmer).

### Data & statistical analysis

For feature selection, LASSO logistic regression model was applied to the 730 texture features extracted from the US images, and a Radiomics Score was calculated for each patient using a linear combination of selected features weighted by the respective coefficients.

Univariable and multivariable logistic regression analysis was performed to calculate the odds ratio with 95% confidence intervals (CI), including patient’s age, gender, tumor size, and radiomics score. For internal validation, bootstrap with 1,000 resampling was used. Calibration curves were plotted to assess the calibration of the model built using the factors included, using the Hosmer Lemeshow test. Harrell’s C-index was measured to evaluate the model’s discrimination ability.

R software (version 3.4.2, http://www.R-project.org) with the R package ‘glmnet’ was used for statistical analysis.

## Results

Among the 527 patients diagnosed with PTC in this study, 428 (81.2%) were positive and 99 (18.8%) were negative for BRAF^V600E^ mutation. Cancer subtype of the 527 masses were proven as follows: 493 (93.5%) as conventional PTC, 23 (4.4%) as follicular variant PTC, 7 (1.3%) as diffuse sclerosing variant PTC, 4 (0.8%) as oncocytic variant of PTC. Demographics according to the presence of BRAF^V600E^ mutations are summarized in Table 1. Mean tumor size was significantly smaller in nodules positive for BRAF^V600E^ mutation, 16.0±7.6 mm to 18.0±9.1 mm (*P* = 0.003). Similar tendency was observed in the training cohort (15.8±7.4 mm to 19.6±10.0 mm, *P* = 0.004), but not in the validation cohort or the conventional PTC <20-mm subgroup.

**Table 1:** Demographic features of the total thyroid cancers and conventional PTCs<20-mm according to the presence of BRAF^V600E^ mutation.

	**Total (n = 527)**								
			***P***	**Training cohort (n = 387)**		***P***	**Validation cohort (n = 140)**		***P***
BRAF^V600E^ mutation	Negative (n = 99)	Positive (n = 428)		Negative (n = 68)	Positive (n = 319)		Negative (n = 31)	Positive (n = 109)	
Mean age (years)	38.4±13.2	42.7±13.8	0.284	38.3±13.4	43.1±13.9	0.009	38.8±13.1	41.7±13.4	0.288
<55 years	86 (86.9%)	334 (78.0%)	0.064	60 (87.0)	247 (77.7)	0.118	27 (87.1)	86 (78.9)	0.446
≥55 years	13 (13.1%)	94 (22.0%)		9 (13.0)	71 (22.3)		4 (12.9)	23 (21.1)	
Gender			0.523			0.997			0.290
Men	79 (79.8%)	326 (76.2%)		15 (22.7)	72 (22.6)		5 (16.1)	30 (27.5)	
Women	20 (20.2%)	102 (23.8%)		54 (78.3)	246 (77.4)		26 (83.9)	79 (72.5)	
Mean size of tumor (mm)	18.0±9.1	16.0±7.6	0.003	19.6±10.0	15.8±7.4	0.004	15.2±7.8	16.2±7.6	0.511
<20mm	67 (67.7%)	341 (79.7%)	0.015	40 (58.0)	254 (79.9)	<0.001	27 (87.1)	87 (79.8)	0.511
≥20mm	32 (32.3%)	79 (20.3%)		29 (42.0)	64 (20.1)		4 (12.9)	22 (20.2)	
Radiomics Score (median, interquartile range)				1.486 (1.160, 1.690)	1.704 (1.501, 1.836)	<0.001	1.615 (1.457, 1.747)	1.672 (1.519, 1.832)	0.238
				**Conventional PTC <20-mm (n = 389)**					
				**Training cohort (n = 280)**		***P***	**Validation cohort (n = 109)**		***P***
				Negative (n = 33)	Positive (n = 247)		Negative (n = 23)	Positive (n = 86)	
Mean age (years)				41.1±13.9	43.3±13.0	0.386	39.5±13.9	43.0±12.6	0.277
<55 years				27 (81.8)	194 (78.5)	0.837	19 (82.6)	68 (79.1)	>0.999
≥55 years				6 (18.2)	53 (21.5)		4 (17.4)	18 (20.9)	
Gender						0.151			0.587
Men				3 (9.1)	53 (21.5)		4 (17.4)	22 (25.6)	
Women				30 (90.9)	194 (78.5)		19 (82.6)	64 (74.4)	
Mean size of tumor (mm)				12.9±2.7	13.0±2.3	0.843	13.1±2.0	13.3±2.4	0.720
<20mm				-	-	--	-	-	-
≥20mm				-	-	--	-	-	-
Radiomics Score (median, interquartile range)				1.887 (1.734, 2.161)	2.117 (1.929, 2.257)	<0.001	2.089 (1.920, 2.246)	2.104 (1.949, 2.223)	0.827

### Feature selection and calcuation of radiomics score

**Fig 2:**
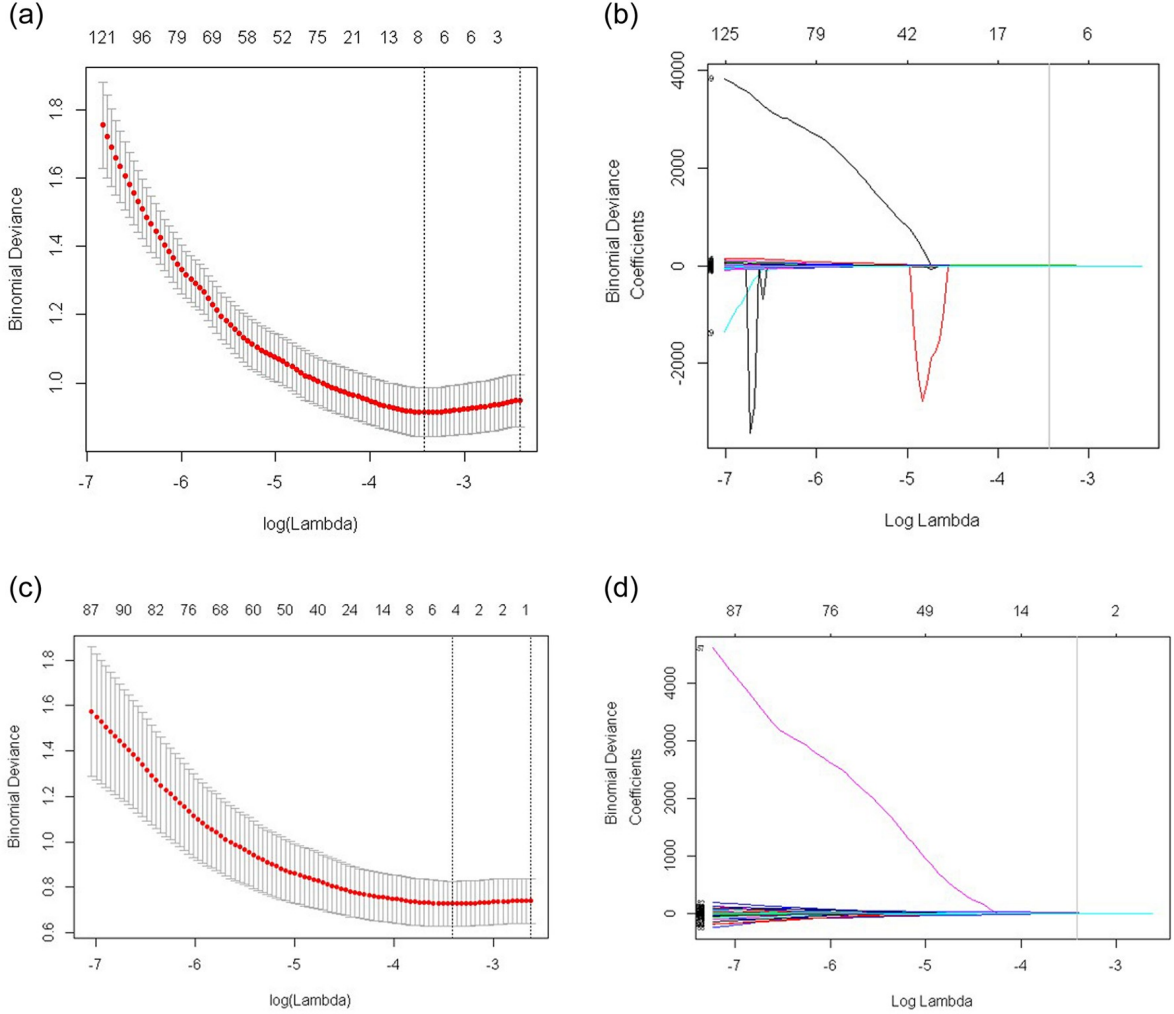
Texture feature selection using the least absolute shrinkage and selection operator (LASSO) logistic regression model. (A) Tuning parameter (lambda, λ) selection in the LASSO model used 10-fold cross validation for 527 thyroid cancers. The mean deviance (goodness-of-fit statistics, red dots) was plotted versus log(λ), error bars displaying the range of standard error. Dotted vertical lines were drawn at the point of minimum deviance (λ value = 0.03229), and at the point where maximum λ was obtained among errors smaller than the standard error of minimum deviance (λ value = 0.08984). (B) LASSO coefficient profiles of the 730 texture features. A coefficient profile was plotted versus log(λ). The gray vertical line was drawn at the value selected using 10-fold cross validation, where the optimal λ resulted in 8 nonzero coefficients. (C) Tuning parameter (lambda, λ) selection in the LASSO model used 10-fold cross validation for 389 conventional PTCs <20-mm. The mean deviance (goodness-of-fit statistics, red dots) was plotted versus log(λ), error bars displaying the range of standard error. Dotted vertical lines were drawn at the point of minimum deviance (λ value = 0.0329208), and at the point where maximum λ was obtained among errors smaller than the standard error of minimum deviance (λ value = 0.072595). (D) LASSO coefficient profiles plotted versus log(λ), gray vertical line was drawn at the value selected using 10-fold cross validation, where the optimal λ resulted in 4 nonzero coefficients.

Eight potential features were selected among 730 texture features in the training cohort with nonzero coefficients in the LASSO logistic regression model (Fig 2A and 2B). These 8 texture features were presented in the calculation formula below used to calculate the Radiomics Score,

$$\begin{array}{l} \underline{\text{Radiomics}\;\text{Score}\;(\text{total})}=0.3715483\;-0.0179227\;\text{X}\;\text{mad}\_6\_0\;-0.0202624\;\text{X}\;\text{sv}\_43\_0\;\\ -0.0000068\;\text{X}\;\text{HL}\_\text{ene}\_1\_0\;-0.0000041\;\text{X}\;\text{HL}\_\text{rln}\_48\_0\;-0.0769504\;\text{X}\;\text{LL}\_\text{uni}\_13\_0\;\\ -0.0013692\;\text{X}\;\text{LL}\_\text{lrlgle}\_54\_0+0.0025444\;\text{X}\;\text{LL}\_\text{se}\_42\_45+0.5554316\;\text{X}\;\text{LL}\_\text{se}\_42\_90 \end{array}$$

For the conventional PTCs measuring <20-mm, 4 potential features were selected among the 730 texture features in the training cohort (Fig 2C and 2D). These 4 texture features were presented in the calculation formula below used to calculate the Radiomics Score (cPTC<20-mm),

$$\begin{array}{l} \underline{\text{Radiomics}\;\text{Score}(\text{cPTC}<20\text{-mm})}=-2.2001791+11.4205518\;\text{X}\;\text{LH}\_\text{srlgle}\_52\_0-0.7666155\;\text{X}\;\text{LL}\_\text{uni}\_13\_0\;\\ +\;0.8461400\;\text{X}\;\text{LL}\_\text{se}\_42\_90-0.0001180\;\text{X}\;\text{LL}\_\text{lrhgle}\_55\_90 \end{array}$$

### Development, performance, and validation of prediction models

Table 2 summarizes the results of univariable and multivariable logistic regression analysis for predicting the presence of BRAF^V600E^ mutations. In the training cohort of the total thyroid cancers, tumor size and Radiomics Score were factors with statistical significance on univariable analysis. Among the training cohort including conventional PTCs measuring <20-mm, Radiomics Score was the single factor showing statistical significance. In both total cancers and the conventional PTC<20-mm, Radiomics Score was the single factor showing significant association to the presence of BRAF^V600E^ mutation on multivariable analysis (all *P*<0.05).

**Table 2:** Univariable and multivariable analysis in predicting the presence of BRAF^V600E^ mutation in the training cohort of the total thyroid cancers and conventional PTC<20-mm.

**Clinical features**	**Total**					
	**Univariable**			**Multivariable**		
	OR	95% CI	*P*	OR	95% CI	*P*
Tumor size	0.953	0.924–0.981	0.001	1.018	0.977–1.060	0.394
Age (≥55 years)	1.916	0.948–4.308	0.089	1.948	0.928–4.536	0.096
Gender	1.054	0.573–2.035	0.871	1.757	0.877–3.793	0.129
Radiomics score	6.099	3.124–12.723	<0.001	8.979	3.603–23.920	<0.001
	**Conventional PTCs<20-mm**					
	OR	95% CI	*P*	OR	95% CI	*P*
Tumor size	1.018	0.875–1.198	0.825	1.030	0.872–1.232	0.738
Age (≥55 years)	1.229	0.512–3.431	0.665	1.282	0.495–3.943	0.632
Gender	2.732	0.926–11.701	0.108	4.281	1.166–27.327	0.060
Radiomics score	9.976	3.161–38.451	<0.001	11.279	3.624–44.121	<0.001

US: ultrasonography, PTC: papillary thyroid carcinoma, OR: Odds ratio, 95% CI: 95% confidence interval

**Fig 3:**
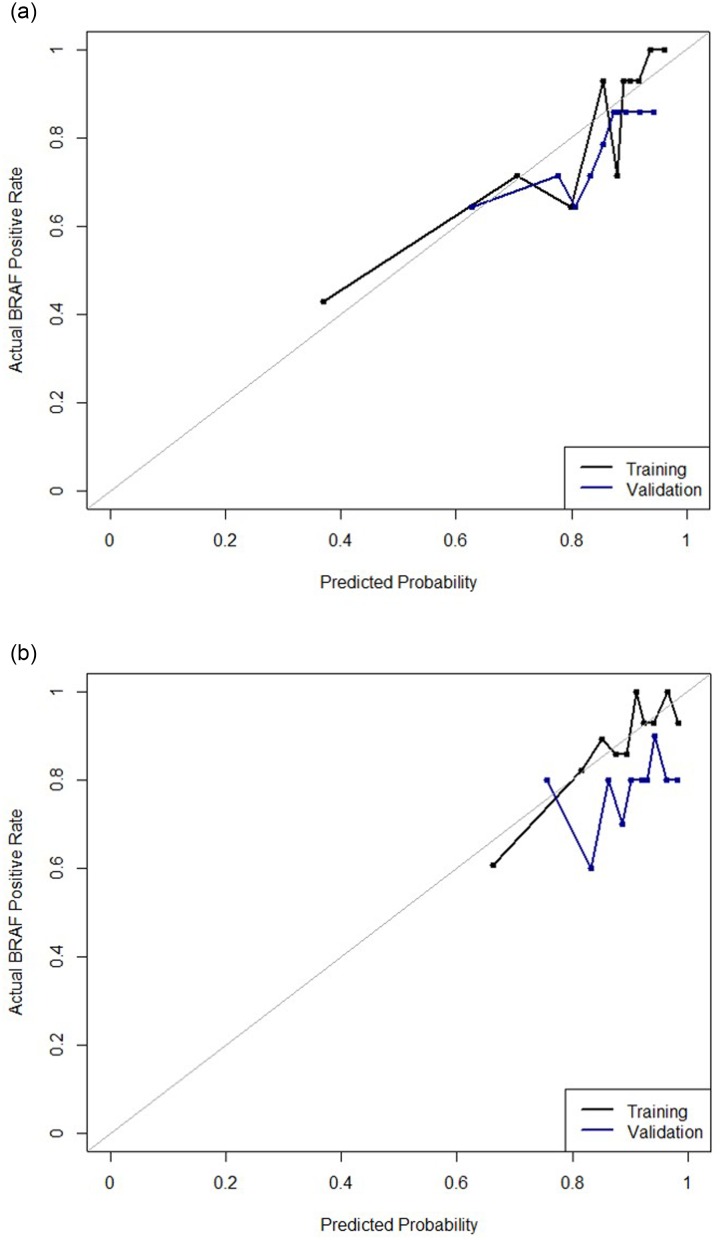
Calibration plots of the grouped prediction models for the presence of BRAF^V600E^ mutation. For each plot, the y-axis represents the actual probability of BRAF^V600E^ mutation, and the x-axis represents the predicted risk for BRAF^V600E^ mutation. (A) Calibration plot for the total thyroid cancers and (B) conventional PTCs measuring <20-mm included in this study.

The calibration curve of the prediction model for the presence of BRAF^V600E^ mutation demonstrated good agreement between prediction and observation in the training cohort among the thyroid cancers. The Hosmer-Lemeshow test yielded statistics of *P* = 0.502, suggesting good calibration (Fig 3A). C-statistics for the training set was 0.718 (95% CI: 0.650–0.786), and 0.629 (95% CI: 0.516–0.742) for the validation set (Table 3). The calibration curve of the prediction model for the presence of BRAF^V600E^ mutation among conventional PTCs <20-mm demonstrated good calibration, with the Hosmer-Lemeshow test yielding statistics of *P* = 0.257 (Fig 3B). C-statistics for training set among the conventional PTCs<20-mm was 0.729 (95% CI: 0.632–0.826), and 0.567 (95% CI: 0.434–0.699) for the validation set.

**Table 3:** Discrimination ability of the models in the total thyroid cancers and the conventional PTCs<20-mm.

			Total (n = 527)			Conventional PTC<20-mm (n = 389)		
	Original		Internal validation		Original		Internal validation	
	c-statistics	95% CI	Boostrapped c-statistics	95% CI	c-statistics	95% CI	Boostrapped c-statistics	95% CI
Training set	0.718	0.650–0.786	0.716	0.652–0.786	0.729	0.632–0.826	0.729	0.634–0.819
Validation set	0.629	0.516–0.742			0.566	0.430–0.701		

## Discussion

One major challenge for thyroid cancer is how to distinguish patients who need aggressive treatment to survive to those who do not. There are no consistent predictors that reliably sorts out aggressive PTCs, and in addition to the lack of prospective data regarding appropriate treatment for PTCs due to its generally excellent survival [9], issues regarding overtreatment for low-risk patients who will not experience PTC-related mortality have surfaced and debated over the recent years. This reflects the need for a more effective and accurate biomarker in predicting aggressive PTCs, including molecular analysis such as BRAF^V600E^ mutations. Mutation analysis requires invasive procedures such as biopsy or surgical resection to retrive specimen to be analyzed. Among the non-invasive imaging biomarkers, radiomics is an emerging method that has the potential to predict molecular characteristics of tumors, using quantitative imaging features extracted using data-characterization algorithms. The most widely used imaging modality in radiomics has been computed tomography (CT) or magnetic resonance imaging (MRI), however, US is the most sensitive and accurate imaging modality for the thyroid which we used in this study.

For feature selection in obtaining a Radiomics Score, the LASSO logistic regression model was used, which enables selecting features based on their strength of association on univariable analysis, and combining the selected features into a radiomics signature [23]. The Radiomics Score obtained was the single factor showing significant association in predicting the presence of BRAF^V600E^ mutation in both univariable and multivariable analysis in the training cohorts (Table 3), showing good discrimination for thyroid cancers with BRAF^V600E^ mutation in the training set (c-statistics 0.718 (95% CI: 0.650–0.786)), but with lower c-statistics for validation set (0.629 (95% CI: 0.516–0.742). Our results show that US-derived radiomics may have potential as a non-invasive biomarker, but currently does not enable accurate prediction of the presence of BRAF^V600E^ mutation in PTCs. There have been other studies proving the potential of US-derived radiomics in predicting disease-free survival or in predicting lymph node metastasis in patients diagnosed with PTC [24,25], but to the best of our knowledge, there are currently no studies using US radiomics features in predicting the presence of BRAF^V600E^ mutations in patients diagnosed with thyroid cancer. Further studies including larger number of cases are anticipated in the future to validate our results.

Among the clinical variables, higher rates of BRAF^V600E^ mutation was seen in thyroid cancers of smaller size, which showed significant association on univariable analysis. As the tumor size and subtype of thyroid cancer has been reported to have association to the presence of BRAF^V600E^ mutation [21], we performed a subgroup analysis using a separate Radiomics Score calculated among the 730 texture features from a subset of 389 thyroid cancers confirmed as conventional PTC<20-mm. When using the Radiomics Score (cPTC<20-mm), similar results were obtained with the total 527 PTCs; Radiomics Score (cPTC<20-mm) was the single factor showing significance on both univariable/multivariable analysis (all *P*<0.001), with c-statistics of 0.729 (95% CI: 0.632–0.826) for the training set, lower values for the validation set, 0.567 (95% CI: 0.434–0.699). This supports that US-radiomics has limited value in predicting BRAF^V600E^ mutation in PTC patients, regardless of size.

There are several limitations to this study. First, as the mutation analysis was performed in a selected group of PTC patients, results of our study does not represent mutation features of the general thyroid cancer population. Second, 81.2% of the PTCs in this study had BRAF^V600E^ mutation analysis, which may have affected our results. PTCs among our population has been known for its high prevalence for BRAF^V600E^ mutation [26], and results may have differed when conducted on different populations. Last, US images were used for feature extraction in obtaining a Radiomics Score that may be used in prediction of the presence of BRAF^V600E^ mutation. Inherent observer variability of US compared to computed tomography (CT) or magnetic resonance imaging (MRI) may have affected our results, but since US is currently the generally applied imaging modality for detecting and differentiating thyroid nodules, feature extraction from US images may be more appropriate in extracting radiomics data among thyroid imaging. Also, ROIs for feature extraction was obtained from one radiologist, and observer variability among different radiologists were not considered in data analysis.

In conclusion, our results show that radiomics features extracted from US has limited value as a non-invasive biomarker for predicting the presence of BRAF^V600E^ mutation status of PTC regardless of size.
